# Clinical and neuroimaging features of familial hemophagocytic lymphohistiocytosis

**DOI:** 10.1007/s00247-025-06454-5

**Published:** 2025-11-18

**Authors:** Mona Gamalludin Alkaphoury, Shaimaa AbdelSattar Mohammad, Noura Bahaa ElDien Farghal, Heba Gomaa Abdelraheem Ali, Iman Ahmed Ragab

**Affiliations:** 1 Department of Diagnostic and Interventional Radiology and Molecular Imaging, Division of Pediatric Radiology, Faculty of Medicine, Ain Shams University, Cairo, 11566 Egypt; 2 Pediatric Hematology Oncology Unit, Children’s Hospital, Faculty of Medicine, Ain Shams University, Cairo, Egypt

**Keywords:** Child, Genetic mutation, Magnetic resonance imaging, Recurrence, Severity of illness index, White matter

## Abstract

**Background:**

Hemophagocytic lymphohistiocytosis is a non-malignant immune regulation disorder, with activation of uncontrolled inflammatory processes and multiorgan damage; primary hemophagocytic lymphohistiocytosis is genetic.

**Objectives:**

To analyze clinical and brain magnetic resonance imaging features in children with familial hemophagocytic lymphohistiocytosis.

**Materials and methods:**

This retrospective study included 28 children with molecularly confirmed hemophagocytic lymphohistiocytosis. Clinical and laboratory manifestations at initial presentation and upon reactivation were recorded. Routine brain magnetic resonance imaging scans were reviewed and severity scores were calculated for different molecular types.

**Results:**

Eleven (39.4%) children presented with neurological symptoms, 13 (46.4%) with developmental delays, and four with altered levels of consciousness. Lesions predominated in white matter (39.3% subcortical, 35.7% periventricular, and 7.1% central), although 25% had gray matter involvement; 78.6% of the cases presented with cerebral volume loss. Brain stem, cerebellar, and meningeal involvement were observed in 14.3%, 25%, and 7.1%, respectively. The most common mutations were in *UNC13D* (53.6%), *PRF* (21.4%), *RAB27A* (17.9%), and *STBXP2* (7.1%); of the children with these mutations, neurological symptoms were observed in 20%, 50%, 80%, and 50%, respectively. Central nervous system reactivation was more prevalent in patients with *RAB27A* mutations (60%). White matter changes were noted in 16.7% of *PRF* cases, predominantly involving central regions, whereas 80% of *RAB27A* cases exhibited periventricular white matter abnormalities. *RAB27A* mutations were associated with higher white matter severity scores, whereas *UNC13D* mutations had higher cerebral atrophy scores.

**Conclusion:**

Variable imaging manifestations were observed in familial hemophagocytic lymphohistiocytosis, with white matter involvement predominating. Patients with *RAB27A* mutations had more frequent clinical and imaging-based neurological involvement.

**Graphical abstract:**

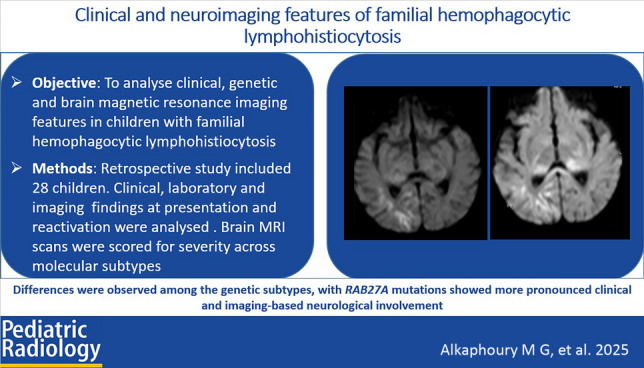

**Supplementary information:**

The online version contains supplementary material available at 10.1007/s00247-025-06454-5.

## Introduction

Hemophagocytic lymphohistiocytosis is an uncontrolled hyperinflammation involving multiple organs. Its features include prolonged fever, hepatosplenomegaly, cytopenia, and central nervous system involvement [1]. However, clinical and genetic diversity poses diagnostic and therapeutic challenges [2]. Hemophagocytic lymphohistiocytosis can be classified as familial or secondary; familial hemophagocytic lymphohistiocytosis most commonly affects infants and children, whereas secondary hemophagocytic lymphohistiocytosis can occur at any age [3]. Familial hemophagocytic lymphohistiocytosis is an autosomal recessive disorder, with mutations in genes involved in cytotoxic granule exocytosis [4].

Hemophagocytic lymphohistiocytosis manifestations include activated lymphocyte and macrophage infiltration into the meninges and brain [5]. Central nervous system (CNS) involvement is present in up to 50% of patients at the time of familial hemophagocytic lymphohistiocytosis diagnosis and may also develop later in the disease course [6].

Brain magnetic resonance imaging (MRI) features of hemophagocytic lymphohistiocytosis are not specific and include patchy areas of abnormal cerebral white matter signal intensity associated with diffuse cerebral and cerebellar parenchymal volume loss [7]. Leptomeningeal and perivascular enhancements corresponding to meningeal and perivascular lymphohistiocytic infiltration have also been documented. Neuro-radiological features may affect the prognosis of familial hemophagocytic lymphohistiocytosis, with restricted diffusion of lesions and numbers of affected brain regions being independent risk factors for death [8, 9].

Few reports have focused on CNS involvement in familial hemophagocytic lymphohistiocytosis and the associated clinical, molecular, and neuroimaging phenotypic features [10]. Our aim was to analyze neurological symptoms and MRI features in children with familial hemophagocytic lymphohistiocytosis in relation to different molecular subtypes, radiological severity, and disease outcomes.

## Materials and methods

### Study population

We retrospectively collected data from 28 children diagnosed with hemophagocytic lymphohistiocytosis at the pediatric hematology-oncology unit of our institute between March 2020 and June 2023. Patients with insufficient medical records were excluded.

Hemophagocytic lymphohistiocytosis diagnostic criteria were based on hemophagocytic lymphohistiocytosis guidelines proposed by Henter et al. [11]. An additional inclusion criterion was a confirmed molecular diagnosis of familial hemophagocytic lymphohistiocytosis. Familial hemophagocytic lymphohistiocytosis genetic screening for consanguineous families included segregation analysis of polymorphic markers in the *perforin* (*PRF1*), *Munc13-4* (*UNC13D*), *syntaxin-11* (*STX11*), and *Munc18-2* (*STXBP2*) genes, followed by Sanger sequencing of the suspected gene. For non-consanguineous families, a dedicated next-generation sequencing panel was used.

The following data were collected: age at onset, disease duration, presenting symptoms and signs, initial complete blood counts, serum ferritin, fibrinogen, fasting triglycerides, bone marrow examination, molecular testing results, frequency of reactivations, and the treatment protocol administered initially and during reactivations. Special emphasis was placed on the neurological manifestations of the original disease and those upon reactivation.

Central nervous system disease was considered positive in the presence of clinical neurological signs and symptoms, including altered levels of consciousness, focal neurological deficits, seizures, and cranial nerve palsies. Neuro-radiological disease was considered positive in the presence of abnormal brain imaging in the absence of other etiologies such as central nervous system infection. Central nervous system disease was defined as the presence of an elevated cerebrospinal fluid white cell count (pleocytosis), elevated protein levels in the absence of detected organisms, or classic autoimmune CNS disease [12].

### Imaging evaluation

All radiological data was retrieved from the hospital picture archiving and communication system.

#### Imaging protocol

All patients underwent brain MRI, using a 1.5-T scanner (Achieva-Philips, Netherlands). A phased array surface coil on the cranial region was used. The field of view was from the vault to the skull base. The following sequences were acquired: axial T1-weighted image (WI) (2–3 mm thickness); axial T2-WI and fluid-attenuated inversion recovery (FLAIR)-weighted images (3 mm thickness); axial diffusion-weighted images (DWI; B=0, B=1,000); and apparent diffusion coefficient images. Contrast media were administered if central nervous system infection was suspected.

#### Image analysis

Brain MRI scans were assessed by two consultant pediatric radiologists with 6 years and 20 years of experience in pediatric imaging; interpretations were made in consensus. Different brain structures (cerebral white matter, gray matter, brain stem, cerebellum, and meninges) were analyzed, and lesion numbers (single, few, or multiple) were recorded. White matter abnormalities were localized in the subcortical, central, or periventricular locations; cortical gray matter and basal ganglia involvement were also assessed. White and gray matter lesions were identified as regions showing T2/FLAIR hyperintensities, T1 hypointensity, or restricted diffusion.

Central nervous system severity scores were used to grade MRI findings based on previously reported grading [13, 14]. White matter severity scores were calculated by adding one point for each affected white matter location (peripheral subcortical, periventricular deep, central, cerebellar hemisphere, cerebellar peduncle, and brain stem); gray matter severity scores were calculated by adding one point for each affected gray matter location (cortical gray matter, basal ganglion, thalamus, dentate nucleus, cerebellar cortical gray matter, dentate nucleus, and brain stem nuclei). Global cerebral atrophy scores were defined as follows: 0, normal; 1, mild-to-moderate (mild-to-moderate dilatation of the cerebral sulci and extra-axial cerebrospinal fluid spaces and/or dilated ventricular system); and 2, severe (dilatation of cerebral sulci with dilated ventricular system).

### Treatment responses

The definitions of response, reactivation, and disease state were based on the 1994 and 2004 hemophagocytic lymphohistiocytosis guidelines [11]. For overall survival, living patients or patients lost to follow-up were censored on the last known survival date.

### Statistical analysis

The collected data were revised, coded, tabulated, and analyzed using SPSS 26.0 (IBM, Armonk, NY). Data were presented and analyzed according to the type of data obtained for each parameter. Descriptive statistics are presented as median and interquartile range (IQR) when the distribution was nonparametric. Group comparisons were performed using the Kruskal–Wallis test for continuous variables and Fisher’s exact test for qualitative variables when the expected count was <5 in >20% of the cells.

## Results

### Patient characteristics

Twenty-eight patients with familial hemophagocytic lymphohistiocytosis were included; 15 (53.6%) had mutations in *UNC13D*, six (21.4%) in *PRF*, five (17.9%) in *RAB27A*, and two (7.1%) in *STXPB2*. The median age at presentation was 14 months (IQR, 6–36; range, 0.5–108). Twenty (71.4%) patients had consanguineous parents, and 10 (64.3%) had a history of sibling death.

Initial CNS disease was present in 10 (33.3%) patients; clinical manifestations included seizures in eight (26.7%), altered levels of consciousness in five (16.7%), ataxia in one (3%), and motor weakness in three (10.7%). Fourteen (46.7%) patients had mental developmental delays; after the induction phase, 10 (33.3%) had residual neurological dysfunction. CNS reactivation occurred in 13 (43.3%) patients. No difference in laboratory parameters was observed between patients with and without CNS disease (Supplementary Material 1).

### Radiological findings

**Fig. 1:**
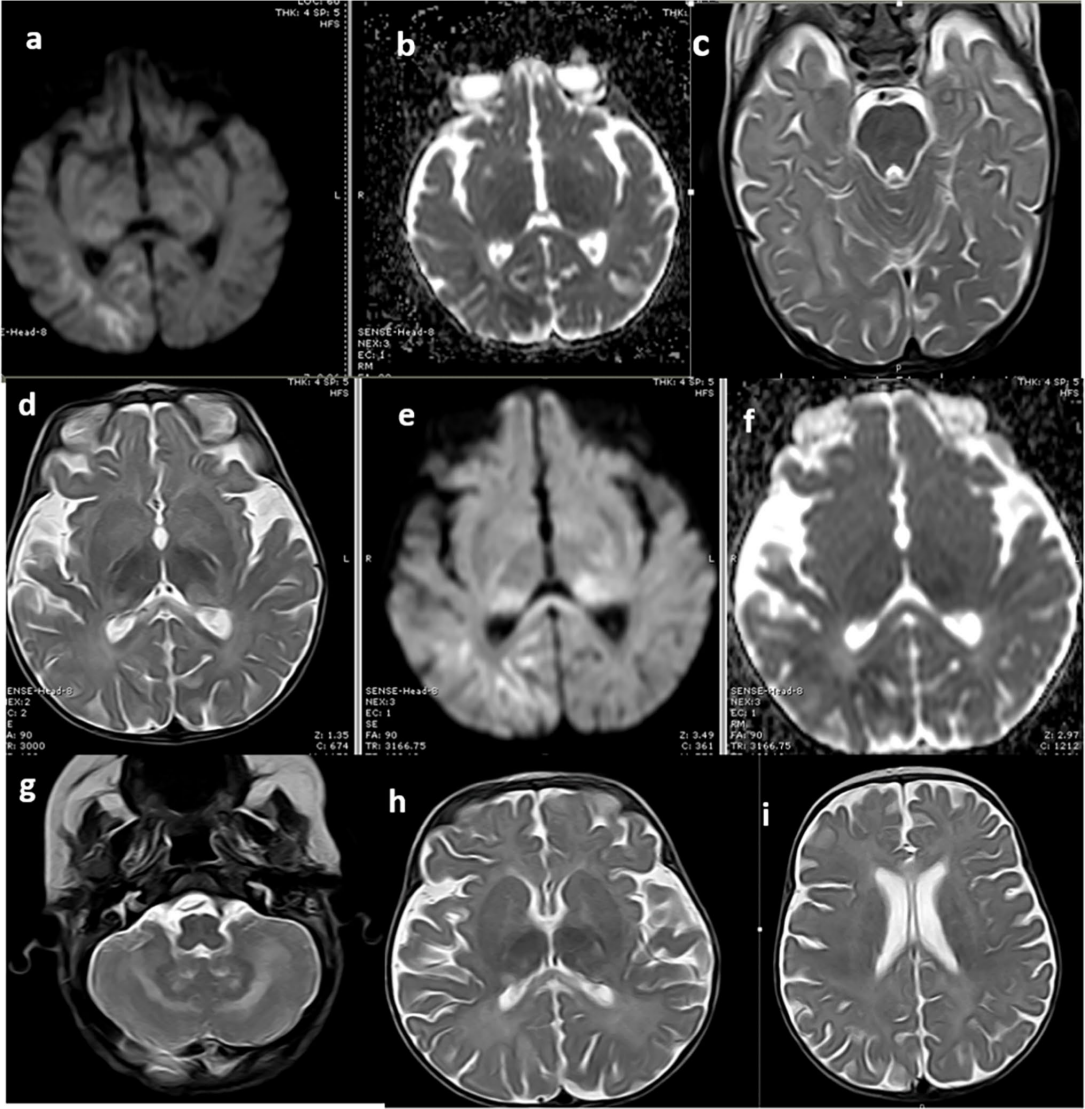
Magnetic resonance imaging manifestations in a female patient with *RAB27A* mutation, presenting with sudden convulsions. **a**, **b**, **c** Axial diffusion-weighted (**a**), apparent diffusion coefficient (**b**), and axial T2-weighted (**c**) images showing right occipital subcortical white matter true diffusion restriction and corresponding high T2 signal intensity. **d**, **e**, **f** Axial T2-weighted (**d**), diffusion-weighted (**e**), and apparent diffusion coefficient (**f**) images showing bilateral asymmetrical posterior thalamic swelling with high T2 signal and true diffusion restriction, more evident on the left side, corresponding with gray matter severity score of 3. **g**, **h**, **i** Axial T2-weighted images at different levels show bilateral symmetrical cerebellar white matter and dentate nuclei patchy high signal in background global atrophic changes, corresponding with a cerebral atrophy severity score of 1, white matter severity score of 3, and total severity score of 7

White matter abnormalities were observed in 17 (60.7%) patients, mostly in the subcortical white matter (28.6%). Regarding the pattern of white matter involvement, nine (32.1%) patients had scattered nonspecific high-signal foci, five (17.9%) had confluent white matter patches, and one (3.6%) had high-signal foci similar to those of small vessel disease. Brain stem abnormalities were found in four (14.3%) patients: two with cerebellar peduncle involvement, one with medullary involvement, and one with both peduncle and pons involvement. Seven (25.0%) patients had lesions in the cerebellum, of which 57.1% had bilateral scattered high T2 signal foci in the subcortical white matter (Fig. 1).

**Fig. 2:**
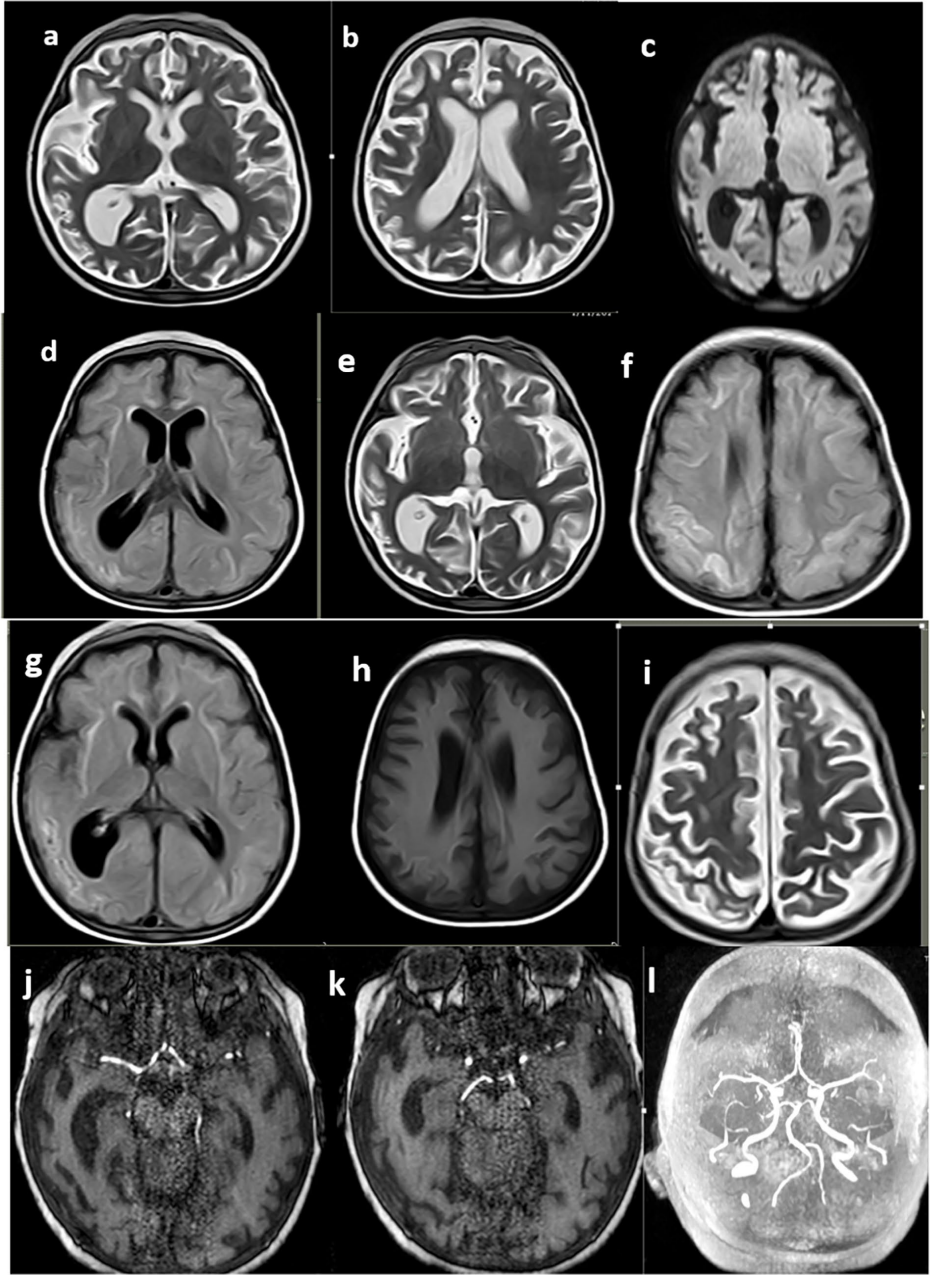
Magnetic resonance imaging manifestations in a male patient with *UNC13D* mutation, presenting with sudden disturbed conscious level and vision loss. **a**, **b** Axial T2-weighted images showing global brain atrophy corresponding with cerebral atrophy severity score of 2. **c** Axial diffusion-weighted image shows no definite true diffusion restriction denoting no acute vascular insult. **d**, **e**, **f** Axial fluid-attenuated inversion recovery (**d**), T2-weighted (**e**), and fluid-attenuated inversion recovery taken more cranially (**f**) images show diffuse cerebral subcortical white matter high signals. **g**, **h**, **i** Axial fluid-attenuated inversion recovery (**g**), T1-weighted (**h**), and T2-weighted (**i**) images show right occipitoparietal areas of gliosis and encephalomalacia with high T1 gyriform signal of cortical laminar necrosis of an old ischemic insult. **j**, **k**, **l** Axial time-of-flight magnetic resonance angiography at the anterior circulation level (**j**), time-of-flight magnetic resonance angiography at the posterior circulation level (**k**), and maximum intensity projection (**l**) images show diffuse irregular luminal narrowing, beading with paucity of terminal arterial arborizations, suggesting small cerebral vessel disease-like patterns

**Fig. 3:**
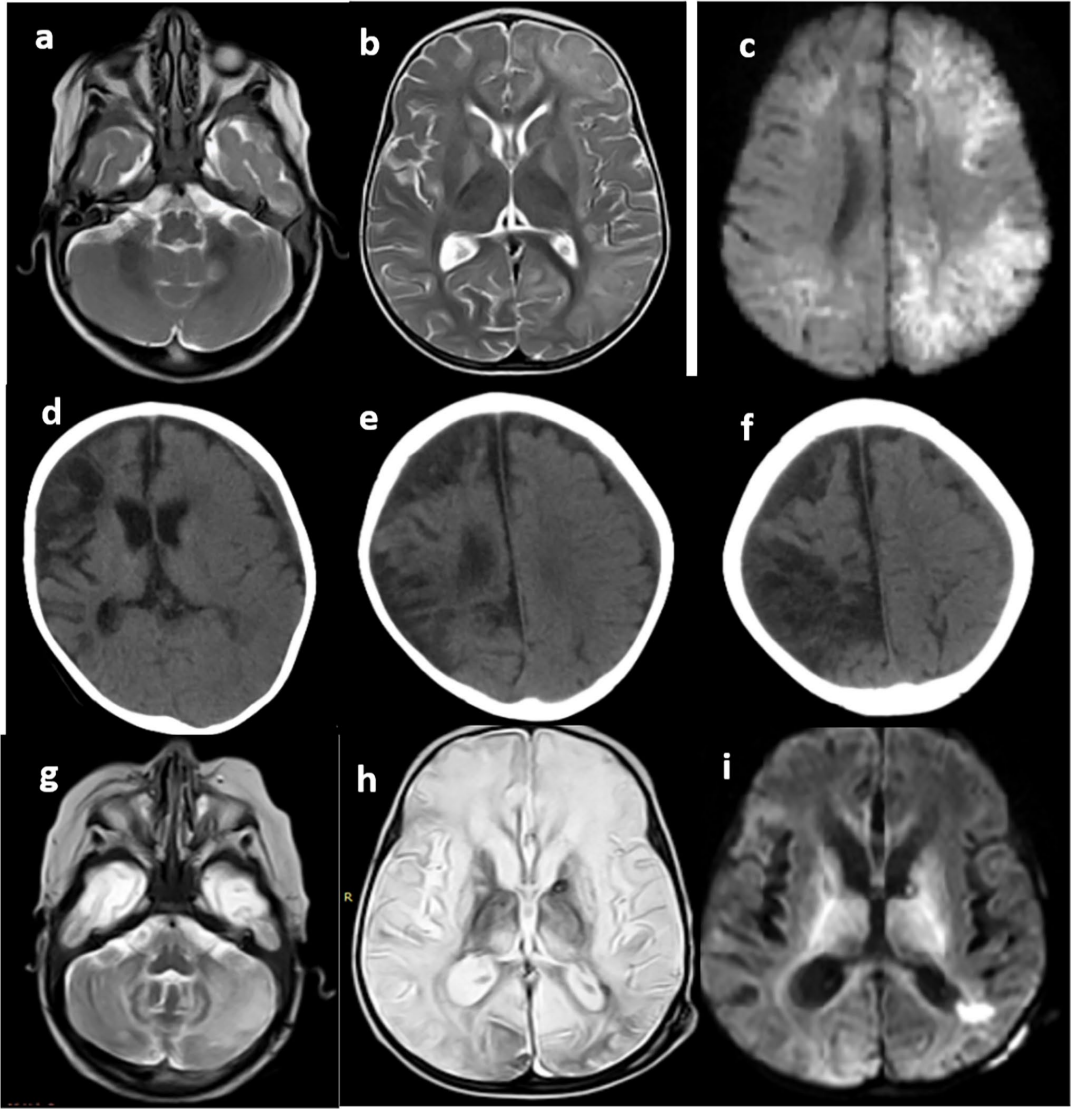
Magnetic resonance imaging manifestations of a patient with *PRF* mutation at initial presentation, follow-up, and central nervous system reactivation. **a**, **b** Initial Magnetic resonance imaging axial T2-weighted images showing left cerebral white matter swelling and high T2 signal with patchy high signal involving the left middle cerebellar peduncle. **c** Axial diffusion-weighted image shows left cerebral patchy high signal of true diffusion restriction. **d**, **e**, **f** Axial computed tomography images at 8-month follow-up show global cerebral atrophic changes with right cerebral gliosis and cystic encephalomalacia. **g**, **h**, **i** Axial T2-weighted image at the cerebellar level (**g**), T2-weighted image at the supratentorial level (**h**), and diffusion-weighted (**i**) images taken at clinical reactivation after 4 months show global white matter gliosis and cerebral atrophy with areas of restricted diffusion

Gray matter signal abnormalities were found in seven (25%) patients, predominantly in the basal ganglia (21.4%); four (66.7%) had bilateral thalamic signal abnormalities, one (3.6%) had right occipital gliosis and cortical laminar necrosis, and one (3.6%) had high T2 signal confluent patches (Figs. 2 and 3).

Twenty-two (78.6%) patients had diffuse cerebral atrophy, which was mild in 50%, moderate in 31.8%, and severe in 9.1%.

Contrast studies were available for nine patients; enhancing patches of white matter abnormalities were found in three (10.7%), and one showed focal gyriform enhancement. Nonspecific foci of diffusion restriction were found in five (17.9%) patients.

Initial MRI severity scores are presented in Table 1. We compared patients with mild total MRI severity scores and those with moderate/severe scores and found that the moderate/severe group showed a higher frequency of clinical central nervous system disease (*P*=0.422), residual neurologic deficit, and CNS reactivation than the mild group (Table 1). Comparisons of laboratory parameters are presented in Supplementary Material 2.

**Table 1:** Clinical criteria and patient outcome in relation to magnetic resonance imaging severity scores in studied patients with familial hemophagocytic lymphohistiocytosis

		Total severity score		*P*-value
		Normal or mild	Moderate or severe	
		*n*=17	*n*=13	
Hemophagocytic lymphohistiocytosis genetic type	***RAB27A***	1 (5.9%)	4 (30.8%)	0.300
	***STBP2***	2 (11.8%)	2 (15.4%)	
	***MUNC13-4***	10 (58.8%)	5 (38.5%)	
	***PRF***	4 (23.5%)	2 (15.4%)	
Initial clinical CNS disease	No initial CNS disease	16 (94.1%)	4 (30.8%)	<0.001
	Initial CNS disease	1 (5.9%)	9 (69.2%)	
Residual neurologic dysfunction and induction	No residual neurocognitive delay	16 (94.1%)	4 (30.8%)	<0.001
	Positive initial neurocognitive delay	1 (5.9%)	9 (69.2%)	
Developmental delay	Negative	14 (82.4%)	2 (15.4%)	<0.001
	Positive	3 (17.6%)	11 (84.6%)	
Outcome	Mortality	9 (52.9%)	11 (84.6%)	0.068
	Survival	8 (47.1%)	2 (15.4%)	
Overall survival (months)	Median (IQR)	22 (5–63)	10 (4.5–13)	0.268
	Range	0.5–113	2–70	
Time from diagnosis to mortality (months)	Median (IQR)	5 (2.5–10)	10 (3–13)	0.368
	Range	0.5–31	2–27	
Systemic reactivation	No systemic reactivation	8 (47.1%)	5 (38.5%)	0.638
	Systemic reactivation	9 (52.9%)	8 (61.5%)	
Duration from diagnosis to reactivation	Median (IQR)	4.5 (0–6)	6 (3–9)	0.194
	Range	0–26	0–18	
Clinical CNS reactivation	No CNS reactivation	14 (82.4%)	3 (23.1%)	0.001
	CNS reactivation	3 (17.6%)	10 (76.9%)	
Number of CNS reactivation	Median (IQR)	0 (0–0)	1 (1–2)	0.003
	Range	0–3	0–5	

*CNS* central nervous system, *IQR* interquartile range, *n* number

Follow-up MRI scans were available for 10 patients, and cerebral atrophy scores were found to be higher upon follow-up (Fig. 3). However, the difference in total severity scores was not statistically significant. No changes in white matter or gray matter severity scores were observed.

### Molecular types analysis

Initial CNS involvement was documented in three (60%) patients with *RAB27A*, one (50%) with *STXBP2*, two (13.3%) with *UNC13D*, and three (50%) with *PRF* mutations. Developmental delays at presentation were observed in all *RAB27A* cases (five patients, 100%), as well as one (50%) *STXBP2*, four (26.7%) *UNC13D*, and three (50%) *PRF* cases, showing a statistically significant difference. Neurological disabilities before the first central nervous system reactivation were observed in two (40%) patients with *RAB27A*, one (50%) with *STXBP2*, three (20%) with *UNC13D*, and three with *PRF* mutations. Regarding MRI findings, central white matter involvement was more frequently observed in patients with *PRF* mutations; periventricular white matter changes were most prominent in those with *RAB27A* mutations (80%), followed by 50% in *PRF*, 20% in *UNC13D*, and none in *STXBP2* cases (Table 2). All patients with *UNC13D* mutations showed periventricular white matter involvement, with volume loss observed in 80% of these cases.

**Table 2:** Comparison of demographic and outcome data among the molecular types of familial hemophagocytic lymphohistiocytosis

Variable		Hemophagocytic lymphohistiocytosis genetic type							
		***RAB27A*** *n*=5		***STBP2*** *n*=2		***UNC13-D*** *n*=15		***PRF*** *n*=6	
		*n*	%	*n*	%	*n*	%	*n*	%
Order of birth	First	2	40.0%	0	0.0%	7	46.7%	1	16.7%
	2–3	2	40.0%	1	50.0%	3	20.0%	2	33.3%
	>3	1	20.0%	1	50.0%	5	33.3%	3	50.0%
Sibling death or affection	Sibling death	2	40.0%	1	50.0%	9	60.0%	6	100.0%
Consanguinity	Positive	5	100.0%	1	50.0%	8	53.3%	6	100.0%
Outcome	Survival	3	60.0%	1	50.0%	3	20.0%	2	33.3%
	Mortality	2	40.0%	1	50.0%	12	80.0%	4	66.7%
BMT	No BMT	3	60.0%	1	50.0%	13	86.7%	5	83.3%
	Underwent BMT	2	40.0%	1	50.0%	2	13.3%	1	16.7%

*BMT* bone marrow transplantations, *n* number

Regarding a dilated ventricular system, eight (80%) patients with *UNC13D* and three (75%) with *RAB27A* mutations had mild ventricular dilatation, whereas none with the other two molecular types had dilatation, cerebral volume loss, or white matter volume or calcification. The distribution of white and gray matter involvement and severity scores across the four molecular subtypes are presented in Table 3. Patients with *RAB27A* mutations had the highest total MRI severity score of 3, primarily due to extensive periventricular white matter lesions. Patients with *PRF*, *UNC13D*, and *STXBP2* mutations had total severity scores of 2, 1, and 0.5, respectively (Table 4).

**Table 3:** Comparison of molecular types according to white and gray matter abnormalities

Imaging abnormalities	Hemophagocytic lymphohistiocytosis genetic type							
	*RAB27A* (*n*=5)		*STBP2* (*n*=2)		*UNC13D* (*n*=15)		*PRF* (*n*=6)	
	*n*	%	*n*	%	*n*	%	*n*	%
Subcortical white matter	2	40.0%	1	50.0%	4	26.7%	4	66.7%
Central white matter	0	0.0%	0	0.0%	1	6.7%	1	16.7%
Periventricular white matter	4	80.0%	0	0.0%	3	20.0%	3	50.0%
Cortical gray matter	0	0.0%	1	50.0%	3	20.0%	2	33.3%
Basal ganglionic gray matter	2	40.0%	0	0.0%	2	13.3%	2	33.3%
Brain stem	3	60.0%	0	0.0%	1	6.7%	0	0.0%
Cerebellum	3	60.0%	0	0.0%	2	13.3%	2	33.3%

*n* number

**Table 4:** Comparison of molecular types according to magnetic resonance imaging severity scores

Radiological findings		Hemophagocytic lymphohistiocytosis genetic type							
		*RAB27A* *n*=5		*STBP2* *n*=2		*UNC13-D* *n*=15		*PRF* *n*=6	
		*n*	%	*n*	%	*n*	%	*n*	%
White matter severity score	0.00	0	0.0%	1	50.0%	9	60.0%	1	16.7%
	1.00	1	20.0%	1	50.0%	4	26.7%	2	33.3%
	2.00	1	20.0%	0	0.0%	1	6.7%	2	33.3%
	3.00	3	60.0%	0	0.0%	0	0.0%	0	0.0%
	4.00	0	0.0%	0	0.0%	0	0.0%	1	16.7%
	6.00	0	0.0%	0	0.0%	1	6.7%	0	0.0%
Gray matter severity score	0.00	3	60.0%	1	50.0%	11	73.3%	4	66.7%
	1.00	1	20.0%	1	50.0%	2	13.3%	0	0.0%
	2.00	0	0.0%	0	0.0%	2	13.3%	2	33.3%
	3.00	1	20.0%	0	0.0%	0	0.0%	0	0.0%
Total severity score	Mild	1	20.0%	0	0.0%	8	61.5%	4	66.7%
	Moderate	3	60.0%	1	100.0%	4	30.8%	1	16.7%
	Severe	1	20.0%	0	0.0%	1	7.7%	1	16.7%

*n* number

### Treatment and outcome

Of the 28 patients analyzed, nine (32.1%) survived, with six having undergone hematopoietic stem cell transplantation. Sixteen (57.1%) patients had systemic reactivation, and 13 (43.3%) had at least one central nervous system reactivation, of which seven (54%) had two or more episodes.

Central nervous system reactivation occurred in seven (50%) patients with *UNC13D*, one (16.7%) with *PRF*, and none (0%) with either *RAB27A* or *STXBP2* mutations (*P*=0.032). Twelve (80%) patients with *UNC13D* mutations died, compared with four (66.7%) with *PRF*, one (50%) with *STXBP2*, and 40% with *RAB27A* mutations (*P*=0.339) (Supplementary Material 3).

**Fig. 4:**
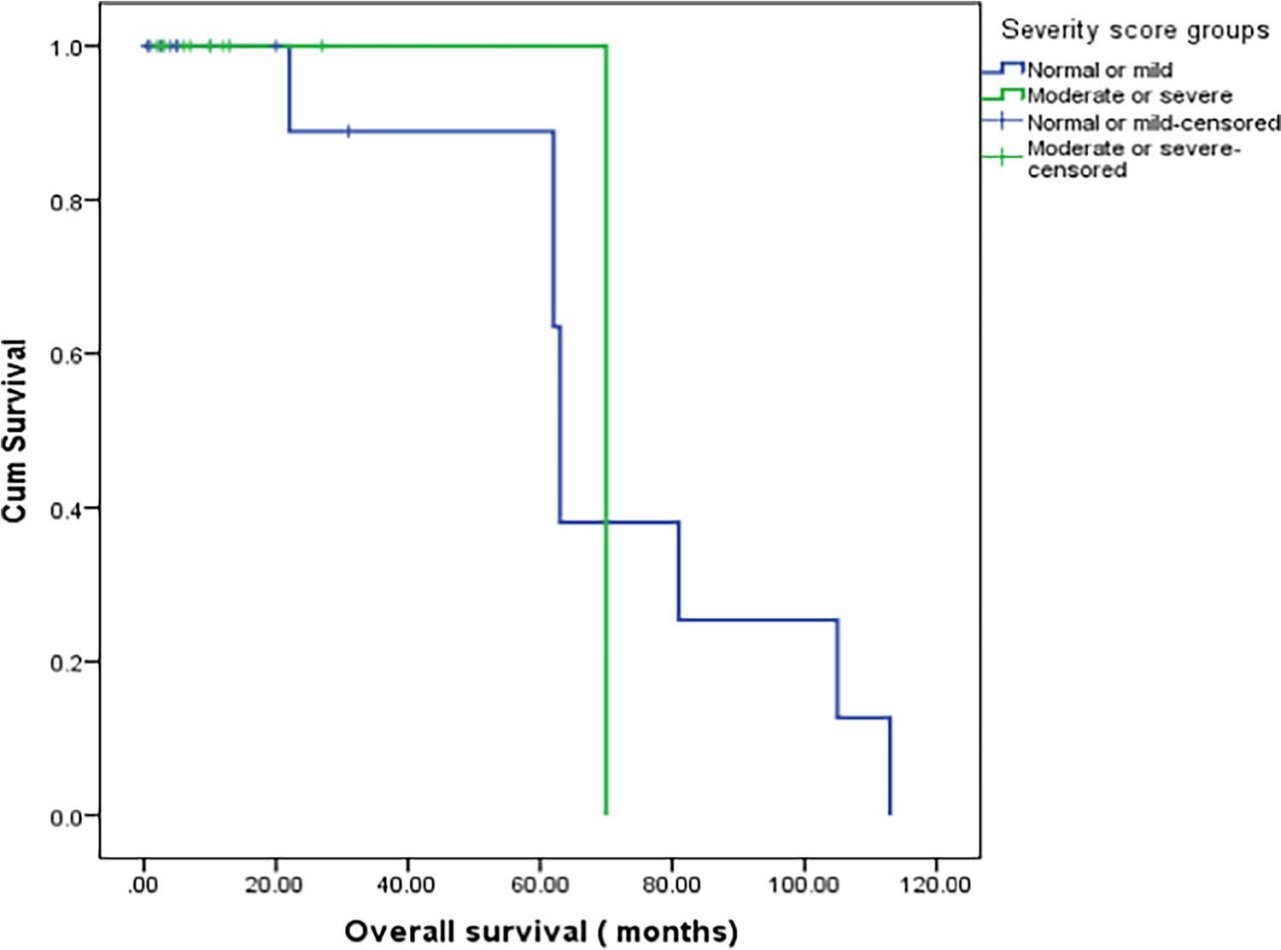
Kaplan–Meier survival curve comparing overall survival according to magnetic resonance imaging (MRI) severity scores. Central nervous system reactivation occurred in 77% of patients with moderate-to-severe radiological abnormalities and 17.6% of those with normal MRI or mild radiological abnormalities. No differences in overall survival and outcomes were observed between groups based on MRI severity scores

Regarding outcomes in relation to MRI severity scores, CNS reactivation occurred in 10 (77%) patients with moderate-to-severe radiological abnormalities and three (17.6%) with normal MRI or mild radiological abnormalities (*P*=0.001); no differences in overall survival were observed between the groups (Fig. 4).

## Discussion

In this study, initial clinical CNS manifestations were detected in over one-third of patients with familial hemophagocytic lymphohistiocytosis. Neurocognitive delays were present in 47% of patients; although mental developmental delays are not a diagnostic criterion of CNS disease in hemophagocytic lymphohistiocytosis, they were found in several patients in the study cohort. Such developmental delays have been previously reported and may improve after bone marrow transplantation [15].

We found no significant differences in the frequency of initial clinical CNS disease based on the molecular subtypes of familial hemophagocytic lymphohistiocytosis, although it was more common in children with *RAB27A* mutations who also showed a significantly higher frequency of mental developmental delays. The frequencies of CNS disease by molecular subgroups vary across different studies. In a European multicenter study of familial hemophagocytic lymphohistiocytosis, the CNS was involved in 60% of patients with *UNC13D* and 36% of those with *PRF* mutations [16]. In a study of patients with *RAB27A* mutation, CNS involvement reached 46% [17]; conversely, a study on familial hemophagocytic lymphohistiocytosis in Saudi Arabia reported no differences in the frequency of CNS involvement between molecular groups, with frequencies of 57% in patients with *STXBP2*, 64% in those with *PRF*, 67% in those with *UNC13D *and *RAB27A* mutations [18].

Generally, clinical CNS manifestations are not limited to a specific mutation in familial hemophagocytic lymphohistiocytosis; in a study of 38 patients with isolated CNS disease as an initial feature, mutations were detected in *PRF* in 23 (61%) patients, *RAB27A* in 10 (26%), *UNC13D* in three (8%), *LYST* in one (3%), and *STXBP2* in one (3%), with a mean duration to diagnosis of 28.3 months [19]. The mechanism of central nervous system manifestations is related to lymphocyte cytotoxicity. At the transient immune synapses between cytotoxic lymphocytes and target cells, *syntaxin-11* binds to *Munc18-2*. Upon receptor activation,* syntaxin-11* is recruited to the synapse, and signals from the activated receptors recruit *Munc13-4* to cytotoxic granules;  *Munc13-4* interacts with *RAB27A* and promotes cytotoxic granule membrane docking. Several previous reports demonstrated a predilection of Griscelli syndrome type 2 in hemophagocytic lymphohistiocytosis [20], with either isolated CNS in 12% or combined CNS and systemic disease in 33% [17]. Although the small number of patients in each molecular subgroup has made interpretation of the frequency of neurological findings difficult, exploring initial CNS disease and its types and frequencies may help explain the predilection of CNS involvement according to molecular types.

Regarding radiological manifestations, cerebral atrophy was the most frequent finding, with 7.1% and 71.4% presenting with severe and mild-to-moderate atrophy, respectively. White matter abnormalities were present in 60.7% of cases, with peripheral subcortical white matter as the primary site of involvement in the form of nonspecific scattered high T2 signal foci. Five (17.9%) patients showed associated white matter nonspecific DWI signal abnormalities and restricted diffusion, with one presenting with an acute ischemic focus on a background of abnormal signal intensity similar to that found in cerebral small vessel disease. Gray matter abnormalities were present in 32% of the patients. Furthermore, although not statistically significant, follow-up imaging revealed increased cerebral atrophy scores.

The reported frequency of neuro-radiological involvement in hemophagocytic lymphohistiocytosis varies across studies. Consistent with our findings, a large series analyzing CNS manifestations in 193 hemophagocytic lymphohistiocytosis cases identified global cerebral atrophy as the most frequent MRI abnormality, followed by white matter changes, demyelination, hemorrhage, calcification, necrosis, and diffuse cerebral edema [21]. Similarly, Deiva et al. identified white matter lesions and cerebral atrophy as the most prevalent imaging features, often accompanied by brainstem and cerebellar involvement [22]. In a study by Ma et al. [23], 17% of children with CNS manifestations had normal MRI scans, 37% demonstrated parenchymal volume loss, and 48% showed brain parenchymal lesions, with notable biochemical differences between the radiological subgroups. Overall, these studies emphasize the variable and multifaceted neuro-radiological presentation of hemophagocytic lymphohistiocytosis [23].

Three different neuropathological stages have been suggested in hemophagocytic lymphohistiocytosis: stage 1, leptomeningeal lymphohistiocytic infiltration; stage 2, additional adjacent parenchymal involvement and perivascular lymphohistiocytic infiltration (with more pronounced meningeal infiltration and involvement of the underlying cortex) [24]; and stage 3, massive lymphohistiocytic infiltration and the presence of tissue necrosis with diffuse infiltration in the brain parenchyma, astrogliosis, white matter and cortical necrosis, and occasional calcifications [25–27]. Whether brain atrophy is necessary for stage 3 diagnosis remains to be determined.

In a study involving seven children with hemophagocytic lymphohistiocytosis, all patients exhibited T2/FLAIR hyperintensities in the supratentorial and infratentorial regions, affecting gray and/or white matter in either nodular or diffuse patterns [28]. Additionally, 86% of the patients showed contrast enhancement involving parenchymal nodules and the cerebral or cerebellar leptomeninges. Cerebral atrophy was present in 57% of cases, often accompanied by white matter signal abnormalities, ventriculomegaly, and enlarged extra-axial cerebrospinal fluid spaces [28]. Similarly, we observed abnormal meningeal and white matter enhancement in our cohort.

We found cerebellar involvement in 25.0% of patients; although cerebellar involvement is not common, a previous study reported marked diffuse cerebellar swelling and obstructive hydrocephalus with mild tonsillar herniation in a child with hemophagocytic lymphohistiocytosis [29]. Regarding white matter diffusion abnormalities, 17.9% of our cohort showed associated white matter nonspecific DWI signal abnormalities and restricted diffusion in patchy and focal patterns, with one patient having acute ischemic changes and acute infarction. These findings align with a previous report of a patient with familial hemophagocytic lymphohistiocytosis type 4 who had an acute infarction that resolved after treatment [30].

In relation to molecular types, all patients with *RAB27A* had white matter involvement, with 60% scoring 3 or higher on the MRI severity score; furthermore, 80% had periventricular white matter involvement compared with 0%, 20%, and 50% in patients with *STXBP2*, *UNC13D*, and *PRF* mutations, respectively. Patients with *PRF* mutations had a higher frequency of moderate white matter involvement compared with the other groups. In a study of isolated CNS diseases in familial hemophagocytic lymphohistiocytosis, diffuse multifocal white matter changes were detected in 79% and cerebellar involvement in 61% of studied patients. *PRF1* mutations were detected in 61%, *RAB27A* in 26%, *UNC13D* in 8%, *LYST* in 3%, and *STXBP2* in 3% [19].

Based on MRI severity scores, CNS reactivation was observed more frequently in patients with moderate/severe initial radiological abnormalities than in those with normal/mild abnormalities, although overall survival did not differ between the groups. In a Chinese study on the association between brain MRI and outcomes, restricted diffusion of lesions and the number of affected brain regions were independent risk factors for death, with an optimal cutoff value of 4.5 affected regions [8].

This study was limited by its relatively small sample size, with few patients representing each molecular subtype, in addition to the inherent constraints of its retrospective design. Consequently, our findings should be validated in larger prospective studies. Nevertheless, we aimed to characterize the clinical and neuro-imaging features of a cohort of genetically diverse children affected by this rare disease. Additionally, the MRI scans were jointly evaluated by two radiologists working in consensus; therefore, the inter-rater agreement was not quantified. Nonetheless, the imaging findings and MRI scores were assessed using well-defined criteria that were reproducible and could be consistently applied by other experienced radiologists.

## Conclusion

We found diverse neuro-imaging patterns in familial hemophagocytic lymphohistiocytosis, with white matter diseases being the most prevalent; molecularly, *RAB27A* mutations were associated with greater clinical and radiological neurological involvement. Patients with moderate-to-severe baseline magnetic resonance imaging abnormalities and higher severity scores were more likely to experience central nervous system reactivation; thus, our results highlight the potential role of severity scoring as a practical tool for comprehensive assessment and risk stratification in familial hemophagocytic lymphohistiocytosis.

## Supplementary information

Below is the link to the electronic supplementary material.

ESM 1 (DOCX 33.1 KB)

## Data Availability

No datasets were generated or analysed during the current study.
