# Supplementary material for: Clinical and neuroimaging features of familial hemophagocytic lymphohistiocytosis
Source: Pediatr Radiol. 2025 Nov 18;56(2):443–54. doi: 10.1007/s00247-025-06454-5 (PMC12881011; doi:10.1007/s00247-025-06454-5)
Supplement: Supplementary file 1 — (DOCX 33.1 KB) [file 247_2025_6454_MOESM1_ESM.docx]

**Supplementary Material 1:**

**Laboratory parameters according to initial neurological manifestations in studied patients with familial hemophagocytic lymphohistiocytosis**

|  | | **Initial CNS disease** | | ***P*-value** |
| --- | --- | --- | --- | --- |
|  |  | **No initial CNS disease** | **Initial CNS disease** |  |
|  |  | ***n*=19** | ***n*=9** |  |
| **Initial ferritin (ng/dl)** | Median (IQR) | 1276 (677 - 6177) | 668 (83 - 1363) | 0.060 |
|  | Range | 194 - 9919 | 1.9 - 2203 |  |
| **Initial TG (mg/dl)** | Median (IQR) | 364 (213 - 487) | 144 (82 - 368) | 0.030 |
|  | Range | 160 - 1380 | 62 - 400 |  |
| **Initial fibrinogen (mg/dl)** | Median (IQR) | 140 (1.7 - 210) | 100 (60 - 180) | 0.954 |
|  | Range | 0.9 - 351 | 0.6 - 300 |  |
| **Initial WBC x 10*9/L** | Median (IQR) | 5.6 (2.3 - 10.7) | 4.5 (3.35 - 6.95) | 0.790 |
|  | Range | 0.5 - 14.2 | 2 - 11.52 |  |
| **Initial NE x 10*9/L** | Median (IQR) | 1 (0.4 - 2) | 0.6 (0.48 - 1.7) | 0.509 |
|  | Range | 0.2 - 4 | 0.3 - 1.8 |  |
| **Initial LY x 10*9/L** | Median (IQR) | 2.6 (1.7 - 6.5) | 2.5 (1.6 - 3) | 0.389 |
|  | Range | 1.2 - 6.9 | 1.4 - 3.4 |  |
| **Initial HB (gm/dl)** | Mean±SD | 8.18 ± 2.18 | 8.71 ± 1.44 | 0.538 |
|  | Range | 3.9 - 11.6 | 7 - 11.2 |  |
| **MCV (fl)** | Mean±SD | 69.64 ± 7.62 | 68.56 ± 10.56 | 0.827 |
|  | Range | 54 - 79 | 57 - 83 |  |
| **Initial PLT x 10*9/L** | Median (IQR) | 61 (34 - 141) | 53 (35.5 - 192) | 1.000 |
|  | Range | 14 - 706 | 23 - 443 |  |
| **Initial ALT (IU/L)** | Median (IQR) | 72 (25 - 105) | 44.5 (15 - 66) | 0.392 |
|  | Range | 8 - 157 | 5 - 164 |  |
| **TBIL (mg/dl)** | Median (IQR) | 1 (0.6 - 6.3) | 0.6 (0.2 - 4.6) | 0.419 |
|  | Range | 0.4 - 13.4 | 0.2 - 4.6 |  |

*ALT* alanine transaminase, *CNS* central nervous system, *HB* hemoglobin, *IQR* interquartile range, *LY* lymphocytes, *MCV* mean corpuscular volume, , *MRI* magnetic resonance imaging, *n* number, *NE* neutrophils, *PLT* platelets, *TBIL* total bilirubin, *TG* triglycerides, *WBC* white blood cells

**Supplementary Material 2:**

**Comparison of laboratory parameters between patients with normal/mild and moderate/severe magnetic resonance imaging severity scores**

|  | | **Total MRI severity score** | | **Test value** | ***P*-value** |
| --- | --- | --- | --- | --- | --- |
|  |  | **Normal or mild** | **Moderate or severe** |  |  |
|  |  | ***n*=16** | ***n*=12** |  |  |
| **Initial ferritin (ng/dl)** | Median (IQR) | 1433.5 (675.5 - 6220) | 1153 (549 - 1363) | -1.370 | 0.171 |
|  | Range | 83 - 9919 | 1.9 - 2203 |  |  |
| **Initial TG (mg/dl)** | Median (IQR) | 375.5 (198 460.5) | 311 (144 - 368) | -1.054 | 0.292 |
|  | Range | 62 - 1380 | 82 - 885 |  |  |
| **Initial fibrinogen (mg/dl)** | Median (IQR) | 144.5 (17.35-224.5) | 95 (60 - 180) | -0.448 | 0.654 |
|  | Range | 0.9 - 351 | 0.6 - 217 |  |  |
| **Initial WBC x 10*9/L** | Median (IQR) | 5.9 (2.3 - 9.55) | 4.5 (4.3 - 9.1) | -0.247 | 0.805 |
|  | Range | 0.5 - 12.2 | 2 - 14.2 |  |  |
| **Initial NE x 10*9/L** | Median (IQR) | 1 (0.4 -2.3) | 0.9 (0.5 - 1.6) | -0.190 | 0.849 |
|  | Range | 0.2 - 4 | 0.3 - 1.8 |  |  |
| **Initial LY x 10*9/L** | Median (IQR) | 2.6 (1.7 - 6.5) | 2.5 (1.6 - 3) | -0.862 | 0.389 |
|  | Range | 1.2 - 6.9 | 1.4 - 3.4 |  |  |
| **Initial HB (gm/dl)** | Mean±SD | 8.17 ± 2.02 | 8.59 ± 1.96 | -0.312 | 0.755 |
|  | Range | 3.9 - 11.6 | 5 - 11.2 |  |  |
| **Initial PLT x 10*9/L** | Median (IQR) | 50 (32.5 -108.5) | 66 (46 - 318) | -1.234 | 0.217 |
|  | Range | 14 - 178 | 23 - 706 |  |  |
| **Initial ALT (IU/L)** | Median (IQR) | 57 (15 -105) | 65.5 (24 - 72) | -0.101 | 0.920 |
|  | Range | 8 - 157 | 5 - 164 |  |  |
| **TBIL (mg/dl)** | Median (IQR) | 0.8 (0.5 - 3.7) | 2.6 (0.6 - 4.6) | -0.264 | 0.792 |
|  | Range | 0.2 - 13.4 | 0.6 - 4.6 |  |  |

*ALT* alanine transaminase, *HB* hemoglobin, *IQR* interquartile range, *LY* lymphocytes*, MRI* magnetic resonance imaging, *n* number, *NE* neutrophils, *PLT* platelets, *TBIL* total bilirubin, *TG* triglycerides, *WBC* white blood cells

**Supplementary Material 3.**

**Individual patient data regarding initial central nervous system disease, reactivation and outcome**

| **Genetic type** | **Initial CNS disease** | **Initial CNS Manifestations** | | **Residual neurologic dysfunction after induction** | **Systemic reactivation** | **CNS reactivation (clinical)** | **Outcome** |
| --- | --- | --- | --- | --- | --- | --- | --- |
|  |  | **Convulsions** | **Altered level of consciousness** |  |  |  |  |
| *RAB27A* | Positive | Positive | Positive | Positive | Positive | Positive | Died |
| *RAB27A* | Positive | Negative | Negative | Negative | Negative | Negative | Survived |
| *RAB27A* | Positive | Positive | Positive | Positive | Positive | Positive | Died |
| *RAB27A** | Negative | Negative | Negative | Negative | Negative | Negative | Survived |
| *RAB27A** | Negative | Negative | Negative | Negative | Negative | Negative | Survived |
| *STXBP2* | Positive | Negative | Negative | Positive | Positive | Positive | Died |
| *STXBP2** | Negative | Negative | Negative | Negative | Negative | Negative | Survived |
| *UNC13D* | Positive | Positive | Negative | Positive | Positive | Positive | Died |
| *UNC13D* | Negative | Negative | Negative | Negative | Positive | Positive | Died |
| *UNC13D* | Negative | Negative | Negative | Negative | Positive | Positive | Died |
| *UNC13D* | Negative | Negative | Negative | Negative | Positive | Positive | Died |
| *UNC13D* | Negative | Negative | Negative | Negative | Negative | Negative | Survived |
| *UNC13D* | Negative | Negative | Negative | Negative | Positive | Positive | Died |
| *UNC13D* | Negative | Negative | Negative | Negative | Positive | Positive | Died |
| *UNC13D** | Negative | Negative | Negative | Negative | Negative | Negative | Survived |
| *UNC13D* | Negative | Negative | Negative | Negative | Positive | Positive | Died |
| *UNC13D* | Negative | Negative | Negative | Negative | Negative | Negative | Died |
| *UNC13D* | Negative | Positive | Negative | Negative | Negative | Negative | Died |
| *UNC13D* | Positive | Positive | Positive | Positive | Positive | Positive | Died |
| *UNC13D* | Negative | Negative | Negative | Negative | Positive | Positive | Died |
| *UNC13D** | Negative | Negative | Negative | Negative | Positive | Positive | Survived |
| *UNC13D* | Negative | Negative | Negative | Negative | Negative | Negative | Died |
| *PRF* | Positive | Positive | Died at initial prolonged presentation | Positive | Negative | Negative | Died |
| *PRF* | Positive | Positive | Positive | Positive | Positive | Positive | Died |
| *PRF* | Positive | Positive | Positive | Positive | Negative | Negative | Died |
| *PRF* | Negative | Negative | Negative | Negative | Negative | Negative | Survived |
| *PRF** | Negative | Negative | Negative | Negative | Positive | Positive | Survived |
| *PRF* | Negative | Negative | Negative | Negative | Positive | Positive | Died |

*CNS central nervous system*
